# Amyloid Beta-Protein and Neural Network Dysfunction

**DOI:** 10.1155/2013/657470

**Published:** 2013-01-30

**Authors:** Fernando Peña-Ortega

**Affiliations:** Departamento de Neurobiología del Desarrollo y Neurofisiología, Instituto de Neurobiología, UNAM, Campus Juriquilla, Boulevard Juriquilla 3001, 76230 Querétaro, Qro, Mexico

## Abstract

Understanding the neural mechanisms underlying brain dysfunction induced by amyloid beta-protein (A*β*) represents one of the major challenges for Alzheimer's disease (AD) research. The most evident symptom of AD is a severe decline in cognition. Cognitive processes, as any other brain function, arise from the activity of specific cell assemblies of interconnected neurons that generate neural network dynamics based on their intrinsic and synaptic properties. Thus, the origin of A*β*-induced cognitive dysfunction, and possibly AD-related cognitive decline, must be found in specific alterations in properties of these cells and their consequences in neural network dynamics. The well-known relationship between AD and alterations in the activity of several neural networks is reflected in the slowing of the electroencephalographic (EEG) activity. Some features of the EEG slowing observed in AD, such as the diminished generation of different network oscillations, can be induced *in vivo* and *in vitro* upon A*β* application or by A*β* overproduction in transgenic models. This experimental approach offers the possibility to study the mechanisms involved in cognitive dysfunction produced by A*β*. This type of research may yield not only basic knowledge of neural network dysfunction associated with AD, but also novel options to treat this modern epidemic.

## 1. Introduction

**Figure 1:**
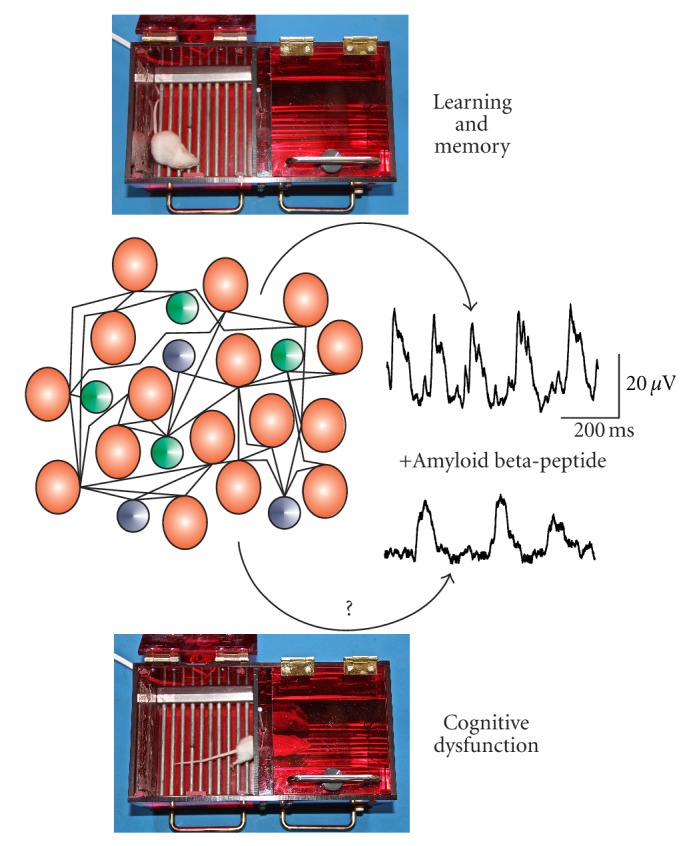
The amyloid beta-peptide disrupts neural network activity along with cognition. The scheme in the middle represents a putative neural network containing neurons with different intrinsic properties (represented in different colors) that interact through synaptic connections (represented by lines). In the case of the hippocampal CA1 network the pyramidal cells, represented by the red circles, interact with each other but also with different populations of GABAergic interneurons, represented by the green and blue circles, to generate different patterns of population activity during cognitive processing. Of course, the generation of oscillatory activity by this network is required during normal cognitive processing (right, upper trace) [23–26, 37], whereas the alterations in such oscillatory activity (lower trace) produced by amyloid beta-protein have been associated with cognitive deficits [20, 21]. The question mark represents the current search for the cellular mechanisms underlying such disruption. The traces on the left are recordings of hippocampal oscillatory activity obtained *in vitro* before and after bath application of amyloid beta-protein 30 nM. The photographs at the top and bottom represent a mouse during a test session of the passive avoidance paradigm. During such a session, control animals tend to remain in the illuminated compartment due to the fact that on the previous day they received an electric shock in the dark compartment. Animals with disrupted memory tend to cross into the dark compartment, as already proven for amyloid beta-application in this paradigm [38, 39]. In summary, the figure represents the relationship between normal cognitive processing and the generation of specific neural network activities as well as the fact that amyloid beta-protein disrupts both of these interconnected processes.

Alzheimer's disease (AD) is a progressive neurodegenerative disorder characterized by severe cognitive impairments [1, 2]. *Postmortem* studies of brains from long-term AD patients have revealed the presence of senile plaques that contain the amyloid beta-peptide (A*β*) [3, 4]. Most studies of AD have focused on the biochemical mechanisms involved in the neurodegenerative processes triggered by the A*β* aggregates (for recent reviews, see [5, 6]). Such efforts have provided noteworthy evidence that has explained some aspects of the disease, mainly in its terminal stages; however, it has been difficult to link these findings to the known cognitive and behavioral symptoms that characterize the early stages of the disease. Moreover, new therapeutic approaches to treat AD based on this research have shown little or no benefit (for a recent review, see [7]). By looking at the cellular mechanisms involved in AD physiopathology from another perspective, it is becoming clear that cognitive decline associated with AD, or with any other neurological disease, should be examined in the context of the related neural network dysfunctions [1, 2, 8–10]. This approach, which might look novel for AD, has had proven success for the understanding of other neurological diseases (e.g., epilepsy; for a recent review, see [11]). One of the main findings supporting this approach in AD is the observation that long before massive neural loss is observed in these patients, there is a significant, early decrease in neuronal activity in various circuits throughout the brain [12, 13], which has also been observed recently in a transgenic mouse model that develops an AD-like pathology [14]. Thus, leaving neurodegeneration aside, we must consider that cognition requires the activity of neural networks (Figure 1) and that knowing how neural network activity is altered in AD will provide a basis to understand the cellular mechanisms of this disease and will allow us to explore new therapeutic avenues against this disease [8–10, 15] (Figure 1).

Over the last several years, evidence has indicated that A*β* is the causal factor for the early cognitive decline observed in AD [1, 2, 8, 9]. Evidence supporting this relationship includes the close correlation between the level of soluble oligomeric forms of A*β* and the cognitive decline in AD patients [3, 4]. Moreover, it has been demonstrated that A*β* acutely disrupts learning and memory after infusion into the CNS [16–19] and that this A*β*-induced cognitive dysfunction can be maintained for long periods of time [20–22]. But, what is the origin of A*β*-induced cognitive dysfunction?

As mentioned, cognition arises from the activity of specific cell assemblies of interconnected neurons that generate neural network dynamics expressed in various patterns of population activity [23–25] (Figure 1). The cellular mechanisms involved in the generation of the different patterns of activity, as well as their specific generators, have been extensively studied in the last decades (for extensive review on this issue look at [23–25]). Of course, these patterns of network activity can be modulated by the intrinsic and synaptic properties of the neurons involved in the circuits in a state-dependent manner (i.e., rest versus active processing; [26]) (Figure 1). Thus, the origin of A*β*-induced cognitive dysfunction must be found in specific alterations in these properties and their consequences in neural network dynamics, as has been explored recently [27–36] (Figure 1).

Several patterns of neural network activities have been linked to specific cognitive processes (Figure 1). For instance, a strong correlation between memory formation and theta rhythm generation has been consistently demonstrated in rodents [24] and humans [40] (Figure 1). Similarly, gamma rhythms have been associated with several cognitive processes [37]. Supporting this association, recent experiments have shown that enhancing gamma activity by optogenetic means increased performance of circuit processing and improved cognition [41], which indicates that the modulation of neural network activity could be used to treat cognitive disorders including AD. Thus, there is evidence that alterations in the generation of neural network activities is involved in several cognitive disorders (for a review, see [41]), including in AD [42–46]. This paper will summarize the evidence regarding the role of A*β* in neural network dysfunction and cognitive decline but will not delve into the possible cellular mechanisms involved since they have been recently reviewed in great detail [1, 2, 5, 6, 8, 9]. Instead, I will highlight the fact that A*β*-induced neural network dysfunction plays a major role in AD and that the study of this process in animal models *in vivo* and *in vitro* can be expected to offer relevant insight into this disease and reveal therapeutic targets against AD-related cognitive decline.

## 2. Alterations in Different Neural Network Patterns Induced by A*β*

Since the generation of different patterns of neural network activity is a prominent feature of several circuits during their involvement in cognitive functions such as memory and learning [23, 24, 47], it is not surprising that alterations in such patterns of activity have been identified in AD patients, whose main manifestation of this disruption is the so-called “EEG slowing” [42–46]. The EEG slowing is observed in the early stages of AD and parallels the cognitive decline observed in these patients [42–46]. Interestingly, similar changes in EEG activity have been observed in transgenic animals that develop an AD-like phenotype through the over-production of A*β* [48–50]. A great deal of evidence points towards a causal role for A*β* in the induction of the neural network dysfunction just described. Experiments from my lab, and others, have shown that some features of the EEG slowing, as well as the cognitive disruption associated with it, can be reproduced by acute application of A*β* in rodents [20–22, 34–36, 51]. However, the evidence obtained from these experiments indicates that the effects of A*β* on the neural network activity are not uniform and, in some rare cases, can be even contradictory. Such “inconsistencies” have also been detected in studies of the oscillatory activity in AD patients. On the one hand, such patients exhibit an increased theta rhythm at rest [42–46], but a reduced induced-theta rhythm during particular cognitive challenges [52]. These observations suggest that the differences in the abnormal neural activity observed in AD as well as the diverse changes in the network activity induced by A*β* may be attributed to the great variety of neural network activity patterns generated by different neural networks throughout the brain and their differential sensitivity to the alterations induced by A*β* [53–55].

## 3. Alterations in Theta and Gamma Rhythms Induced by A*β*

As mentioned, theta rhythm oscillations have been closely related to different cognitive processes both in rodents [24] and humans [40]. Several groups, including ours, have reported that a single intracerebral injection of A*β* induces an acute as well as a long-term reduction in theta rhythm generation [20–22, 34–36], which in turn induces cognitive dysfunction [20–22]. Moreover, we and others have taken this finding a step further and have shown that acute application of A*β* *in vitro* induces a reduction in various neural network patterns including theta rhythm [51, 53, 56, 57]. In agreement with these findings, several transgenic mice that overproduce A*β*, and that exhibit cognitive decline, have shown alterations in theta rhythm generation [14, 48–50, 58]. In transgenic mice expressing the amyloid precursor protein containing the Swedish mutations (K670N, M671L; APP_swe_), a higher theta/delta ratio was found during the non-REM period of the sleep-wake cycle [58]. The double transgenic mouse expressing APP_swe_ and mutated presenilin 1 (A246E) show enhanced theta rhythm during wakefulness and REM sleep [49], an observation that was reproduced, for the theta rhythm during REM sleep, by the same group in other AD transgenic mice that expressed the APP containing the Swedish and London mutations (V717I), the mutated presenilin 1 (A246E), as well as the TAU protein double mutant P301L and R406W, called the PLB1triple transgenic mouse [14]. In contrast, the double transgenic mouse carrying the APP_swe_ and presenilin 1 (G384A) mutations showed an age-dependent decrease in theta hippocampal activity elicited by brainstem stimulation [50]. Similarly, a significant reduction in theta oscillations was observed in other double transgenic mice carrying the APP697 mutations K595N and M596L as well as the mutated presenilin 1 (A246E) [48]. To my knowledge, the first indication that A*β* induces alterations in theta rhythm generation in rodents was reported by Sun and Alkon [27], who found that intracerebroventricular injection of A*β* induces cognitive decline without affecting synaptic transmission or long-term potentiation. However, they observed that the hippocampus of A*β*-treated animals cannot generate carbachol-induced theta oscillations *ex vivo *[27]. Similarly, a reduction in carbachol-induced theta rhythm was found in hippocampal slices taken from the triple transgenic mice that express APPswe, the mutated presenilin 1 (M146V), and the mutated TAU (P301L) [59]. An identical finding has been reported for gamma rhythm in transgenic APP_swe_ mice [28].

As mentioned before, gamma rhythms have also been associated with several cognitive processes, and their disruption is associated with several neurological disorders, including AD (for a review, see [37]). Besides the finding that APP_swe_ mice express a reduction in the generation of kainate-induced gamma rhythm *ex vivo* [28], other alterations in gamma rhythm generation have been found in animal models of AD. For instance, the transgenic mouse that expresses APP with the Swedish and Indiana mutations (V717F) exhibits lower spontaneous gamma activity in the hippocampus *in vivo* [60]. However, other authors have found that the APP_swe_ transgenic mice show no alterations in either the fast oscillations (ripples) or in the sharp waves where they are superimposed [61]; indeed, gamma oscillations were even found to increase in the double transgenic mouse carrying the APP697 mutations K595N and M596L as well as the mutated presenilin 1 (A246E) [48]. Interestingly, in addition to altered hippocampal gamma oscillations related to A*β* presence, a close correlation between reduced gamma activity and a functional behavioral deficit was recently detected in the olfactory network of the APP_swe_ transgenic mouse [62]. The same transgenic mouse exhibit an early increase in olfactory bulb gamma oscillations that correlates with an increase of gamma oscillatory activity in the piriform cortex. However, such early “hyperexcitability” leads the olfactory network into a hyporesponsive state that correlates with a reduction in gamma oscillations in the piriform cortex [63]. Finally, also in the cortex, we found recently that A*β* reduces the power of beta-gamma bursts produced by the entorhinal cortex *in vitro* [36].

The complex changes observed in different oscillatory activities in AD pathology as well as the complex effects that A*β* exerts on them can be explained by the fact that such oscillations are not homogeneous; instead, they represent a broad variety of network functional configurations that rely on complex mixtures of intrinsic and synaptic properties [54, 55, 64]. Rather than constituting a disadvantage, the differential effects that AD pathology and A*β* have on the diverse oscillatory patterns, along with a thorough characterization of such relationships, would reveal key network properties affected by A*β* that would be potential therapeutic targets [53].

## 4. Other Alterations in Neural Population Activity Induced by A*β*

Besides electrophysiological means, neural network activity can also be analyzed through functional multi-neuron calcium imaging, which allows the evaluation of neural network dynamics with single-cell resolution [34, 65]. Using this approach, it has been found that medial septal neurons lose their theta firing coherence upon A*β* application [66]. This effect has been evaluated in cultured neurons that exhibit synchronous spontaneous calcium transients [67–70], showing that either increasing A*β* production by transfecting the cultures with the human APP gene [69] or by directly applying A*β* to the culture medium drastically reduced the synchronized neuronal calcium oscillations [67, 68, 70]. Recently, calcium imaging has also been used *in vivo* to evaluate neural activity, either in the hippocampal CA1 region or in the cortex of the double transgenic mice expressing APPswe and mutated presenilin 1 (G384A) [30, 31]. These studies revealed that neural networks located in the proximity of “senile plaques” are profoundly disturbed and exhibit both an increase in the number of silent neurons as well as an increased number of hyperactive ones [30, 31]. Interestingly, in one of these studies, the direct application of A*β* induced an increase in neuronal calcium transients that lasted for few seconds [31]. In contrast, in our hands, application of A*β* to hippocampal slices induced a reduction in the number of cells that exhibited calcium transients within a few minutes. The neurons that remained active in the presence of A*β* showed a frequency of calcium transients similar to that in control conditions [34].

Patch clamp recordings have demonstrated that A*β* disrupts synchronized synaptic activity in the prefrontal cortex depending on the concentration of the peptide and the duration of application [33]. Application of a low concentration of A*β* (1 nM) inhibits synchronized activity, whereas application of a higher concentration of A*β* (500 nM) induced a biphasic effect that consisted of an initial decrease in network activity followed by an overexcitation [33]. An opposite finding was observed in neural networks cultured on multielectrode arrays, where A*β* application can produce an acute and transient reduction in neural network activity. However, if A*β* exposure is maintained for several hours (24 h) the A*β*-induced inhibition of neural network activity is reversed, and the activity becomes indistinguishable from the control [71]. All these findings clearly show that the effects of A*β* on neuronal network activity can be time and concentration dependent. It is possible that during prolonged A*β* exposure the peptide loses its ability to inhibit neural network activity through enzymatic degradation or sequestration into plaques. On the other hand, A*β* could lead to differential changes in neural network activity (even overexcitation) by forming aggregates with different sizes that produce differential effects on network activity [53]. Alternatively, it is possible that neural networks can adapt their activity to the presence of A*β* by changing their properties to compensate for the inhibitory effects produced by A*β*. In fact, in some cases, deregulation of such compensatory changes could lead to the generation of aberrant hyperexcitable states, such as those observed in several AD transgenic mice. 

## 5. Induction of “Aberrant Activity” by A*β*

Some reports that characterized the EEG activity throughout the sleep-wake transitions in certain strains of AD transgenic mice found no evidence for epileptiform activity [14, 49, 58]; however, other long-term EEG recordings of several lines of AD transgenic mice have revealed spontaneous, nonconvulsive epileptiform discharges that, in some cases, contributed to sudden death in these animals [32, 72–74]. The generation of epileptiform activity has also been correlated with cognitive decline in several of these transgenic mice [60, 72, 73]. Interestingly, recent findings have shown that the epileptiform activity emerges during periods of reduced gamma oscillatory activity and that both the epileptiform activity as well as the cognitive deficits reported, in a transgenic mouse that expresses the APP with the Swedish and Indiana mutations (V717F), are corrected when gamma activity is re-established by genetic means [60]. In contrast, another recent study reported that the epileptiform activity observed in the double transgenic mouse expressing APP_swe_ and presenilin 1 with deleted exon 9 correlates with increased fast oscillatory activity in the thalamocortical network [75]. 

In contrast to the evidence just reviewed, there is other evidence indicating that, instead of having a proepileptic effect, A*β* may indeed reduce epileptiform activity. For example, it has been found that slices taken from APP_swe_ transgenic mice have a reduced frequency of epileptiform synchronous events induced by 4-aminopyridine [76], which is a strong proconvulsant both *in vivo* and *in vitro* [77–79]. Moreover, A*β* was shown to reduce epileptiform activity induced *in vitro* by chronic blockade of GABAergic inhibition [80]. Again, the explanation for these different effects of A*β* on distinct neural network activities can be found in the diversity of epileptiform states that networks can evolve into or in the various compensatory changes induced by the presence of A*β*.

## 6. Conclusions

The data summarized in this paper support the notion that a major component of A*β*-induced cognitive decline is the alteration of diverse neural network activity patterns. The experimental findings described here clearly indicate that the EEG slowing observed in AD patients can be reproduced both *in vivo* and *in vitro* in animal models of AD, which represent an excellent opportunity to study the cellular mechanisms involved in cognitive decline as a way to reveal therapeutic targets for AD. Of course, the evidence shows that the effects of A*β* on neural network activity are rather complex and depend on its concentration and conformation, as well as the duration of its application. However, such complexity, if well characterized, would provide evidence of specific cellular mechanisms affected by A*β* that would be essential for most, if not all, of the disturbances of neural network activity produced by this peptide. It is likely that several of the seemingly contradictory A*β*-dependent effects represent different elements of the same causal chain or, alternatively, that they represent independent branches of a more complex pathogenic process. Since A*β* produces a strong deleterious effect on neural networks, it is likely that several strategies would develop to compensate for the inhibition produced by A*β* and that, in some cases, the failure of such adaptive changes would lead some networks to more disruptive states (hyperexcitation). Of course, it would be essential to determine which of the diverse effects of A*β* on neural network activity account for the cognitive dysfunction observed both in animal models and in AD patients.

Finally, the study of A*β*-induced neural network dysfunction offers an important, alternative view for the understanding of AD pathology. This pathological process, which does not necessarily involve neurodegeneration in its early stages, would provide an experimental model to test pharmacological or nonpharmacological means to prevent such network disruption. For instance, it has been shown that reestablishing gamma oscillation by overexpression of the Nav1.1 channel reduces the aberrant epileptiform activity and the cognitive decline in the transgenic mouse expressing the APP with the Swedish and Indiana mutations [60]. Similarly, the normalization of the EEG in the APP_swe_ transgenic mouse, by passive A*β* immunization, correlates with a reduction in the circadian rhythm alterations observed in these mice [58]. Moreover, the reduction in the epileptiform activity with several antiepileptic drugs reduced cognitive dysfunction in AD transgenic mice [74, 81]. It has also been shown that lowering arachidonic acid levels by inhibiting the activity of group IVA phospholipase A2 reduced the effect of A*β* on neural network activity and prevented A*β*-dependent cognitive deficits in transgenic AD mice that expresses the APP with the Swedish and Indiana mutations [82]. Finally, we have recently reported that the inhibition of GSK3 either with a specific inhibitor or with lithium, which is already in clinical use for the bipolar-disorder [83], abolishes the inhibitory effect of A*β* on the generation of beta-gamma activity in the entorhinal cortex. This and other observations support the use of lithium in the treatment of AD [57]. These are just some examples of the promising venue that has been opened by investigations of neural network disturbances induced by A*β*. Whether or not these studies will render therapeutic approaches to treat AD, remains to be determined.

## References

[1] Selkoe D. J. (2002). Alzheimer's disease is a synaptic failure. *Science*.

[2] Walsh D. M., Selkoe D. J. (2007). A*β* oligomers: a decade of discovery. *Journal of Neurochemistry*.

[3] Lue L. F., Kuo Y. M., Roher A. E. (1999). Soluble amyloid *β* peptide concentration as a predictor of synaptic change in Alzheimer's disease. *American Journal of Pathology*.

[4] Näslund J., Haroutunian V., Mohs R. (2000). Correlation between elevated levels of amyloid *β*-peptide in the brain and cognitive decline. *Journal of the American Medical Association*.

[5] Benilova I., Karran E., De Strooper B. (2012). The toxic A*β* oligomer and Alzheimer's disease: an emperor in need of clothes. *Nature Neuroscience*.

[6] Sheng M., Sabatini B. L., Südhof T. C. (2012). Synapses and Alzheimer's disease. *Cold Spring Harbor Perspectives in Biology*.

[7] Corbett A., Smith J., Ballard C. (2012). New and emerging treatments for Alzheimer's disease. *Expert Review of Neurotherapeutics*.

[8] Palop J. J., Mucke L. (2010). Amyloid-*β*-induced neuronal dysfunction in Alzheimer's disease: from synapses toward neural networks. *Nature Neuroscience*.

[9] Wesson D. W., Nixon R. A., Levy E., Wilson D. A. (2011). Mechanisms of neural and behavioral dysfunction in Alzheimer's disease. *Molecular Neurobiology*.

[10] Peña F., Gutiérrez-Lerma A. I., Quiroz-Baez R., Arias C. (2006). The role of *β*-amyloid protein in synaptic function: implications for Alzheimer's disease therapy. *Current Neuropharmacology*.

[11] Faught E. (2012). Antiepileptic drug trials, the view from the clinic. *Epileptic Disorders*.

[12] Rombouts S. A. R. B., Goekoop R., Stam C. J., Barkhof F., Scheltens P. (2005). Delayed rather than decreased BOLD response as a marker for early Alzheimer's disease. *NeuroImage*.

[13] Prvulovic D., van de Ven V., Sack A. T., Maurer K., Linden D. E. J. (2005). Functional activation imaging in aging and dementia. *Psychiatry Research*.

[14] Platt B., Drever B., Koss D. (2011). Abnormal cognition, sleep, EEG and brain metabolism in a novel knock-in Alzheimer mouse, PLB1. *PLoS ONE*.

[15] Small D. H. (2008). Network dysfunction in Alzheimer's disease: does synaptic scaling drive disease progression?. *Trends in Molecular Medicine*.

[16] Cleary J. P., Walsh D. M., Hofmeister J. J. (2005). Natural oligomers of the amyloid-*β* protein specifically disrupt cognitive function. *Nature Neuroscience*.

[17] Lesné S., Ming T. K., Kotilinek L. (2006). A specific amyloid-*β* protein assembly in the brain impairs memory. *Nature*.

[18] Balducci C., Beeg M., Stravalaci M. (2010). Synthetic amyloid-*β* oligomers impair long-term memory independently of cellular prion protein. *Proceedings of the National Academy of Sciences of the United States of America*.

[19] Kittelberger K. A., Piazza F., Tesco G., Reijmers L. G. (2012). Natural amyloid-beta oligomers acutely impair the formation of a contextual fear memory in mice. *PLoS ONE*.

[20] Mugantseva E. A., Podolski I. Y. (2009). Animal model of Alzheimer's disease: characteristics of EEG and memory. *Central European Journal of Biology*.

[21] Villette V., Poindessous-Jazat F., Simon A. (2010). Decreased rhythmic GABAergic septal activity and memory-associated *θ* oscillations after hippocampal amyloid-*β* pathology in the rat. *Journal of Neuroscience*.

[22] Villette V., Poindessous-Jazat F., Bellessort B. (2012). A new neuronal target for beta-amyloid peptide in the rat hippocampus. *Neurobiology of Aging*.

[23] Traub R. D., Spruston N., Soltesz I., Konnerth A., Whittington M. A., Jefferys J. G. R. (1998). Gamma-frequency oscillations: a neuronal population phenomenon, regulated by synaptic and intrinsic cellular processes, and inducing synaptic plasticity. *Progress in Neurobiology*.

[24] Buzsáki G. (2002). Theta oscillations in the hippocampus. *Neuron*.

[25] Klausberger T., Somogyi P. (2008). Neuronal diversity and temporal dynamics: the unity of hippocampal circuit operations. *Science*.

[26] Ramirez J. M., Tryba A. K., Peña F. (2004). Pacemaker neurons and neuronal networks: an integrative view. *Current Opinion in Neurobiology*.

[27] Sun M. K., Alkon D. L. (2002). Impairment of hippocampal CA1 heterosynaptic transformation and spatial memory by *β*-amyloid 25–35. *Journal of Neurophysiology*.

[28] Driver J. E., Racca C., Cunningham M. O. (2007). Impairment of hippocampal gamma (*γ*)-frequency oscillations in vitro in mice overexpressing human amyloid precursor protein (APP). *European Journal of Neuroscience*.

[29] Cacucci F., Yi M., Wills T. J., Chapman P., O'Keefe J. (2008). Place cell firing correlates with memory deficits and amyloid plaque burden in Tg2576 Alzheimer mouse model. *Proceedings of the National Academy of Sciences of the United States of America*.

[30] Busche M. A., Eichhoff G., Adelsberger H. (2008). Clusters of hyperactive neurons near amyloid plaques in a mouse model of Alzheimer's disease. *Science*.

[31] Busche M. A., Chen X., Henning H. A. (2012). Critical role of soluble amyloid-*β* for early hippocampal hyperactivity in a mouse model of Alzheimer's disease. *Proceedings of the National Academy of Sciences of the United States of America*.

[32] Minkeviciene R., Rheims S., Dobszay M. B. (2009). Amyloid *β*-induced neuronal hyperexcitability triggers progressive epilepsy. *Journal of Neuroscience*.

[33] Wang Y., Zhang G., Zhou H., Barakat A., Querfurth H. (2009). Opposite effects of low and high doses of A*β*42 on electrical network and neuronal excitability in the rat prefrontal cortex. *PLoS ONE*.

[34] Peña F., Ordaz B., Balleza-Tapia H. (2010). Beta-amyloid protein (25-35) disrupts hippocampal network activity: role of Fyn-kinase. *Hippocampus*.

[35] Colom L. V., Castañeda M. T., Bañuelos C. (2010). Medial septal *β*-amyloid 1-40 injections alter septo-hippocampal anatomy and function. *Neurobiology of Aging*.

[36] Peña-Ortega F., Bernal-Pedraza R. (2012). Amyloid beta peptide slows down sensory-induced hippocampal oscillations. *International Journal of Peptides*.

[37] Uhlhaas P. J., Singer W. (2006). Neural synchrony in brain disorders: relevance for cognitive dysfunctions and pathophysiology. *Neuron*.

[38] Bagheri M., Joghataei M. T., Mohseni S., Roghani M. (2011). Genistein ameliorates learning and memory deficits in amyloid *β*(1-40) rat model of Alzheimer's disease. *Neurobiology of Learning and Memory*.

[39] Nobakht M., Hoseini S. M., Mortazavi P. (2011). Neuropathological changes in brain cortex and hippocampus in a rat model of Alzheimer's disease. *Iran Biomedical Journal*.

[40] Lega B. C., Jacobs J., Kahana M. (2012). Human hippocampal theta oscillations and the formation of episodic memories. *Hippocampus*.

[41] Sohal V. S., Zhang F., Yizhar O., Deisseroth K. (2009). Parvalbumin neurons and gamma rhythms enhance cortical circuit performance. *Nature*.

[42] Kowalski J. W., Gawel M., Pfeffer A., Barcikowska M. (2001). The diagnostic value of EEG in Alzheimer disease: correlation with the severity of mental impairment. *Journal of Clinical Neurophysiology*.

[43] Schreiter-Gasser U., Gasser T., Ziegler P. (1994). Quantitative EEG analysis in early onset Alzheimer's disease: correlations with severity, clinical characteristics, visual EEG and CCT. *Electroencephalography and Clinical Neurophysiology*.

[44] Nobili F., Copello F., Vitali P. (1999). Timing of disease progression by quantitative EEG in Alzheimer's patients. *Journal of Clinical Neurophysiology*.

[45] Ihl R., Dierks T., Martin E. M., Frölich L., Maurer K. (1996). Topography of the maximum of the amplitude of EEG frequency bands in dementia of the Alzheimer type. *Biological Psychiatry*.

[46] Babiloni C., Frisoni G. B., Pievani M. (2009). Hippocampal volume and cortical sources of EEG alpha rhythms in mild cognitive impairment and Alzheimer disease. *NeuroImage*.

[47] Harris K. D., Csicsvari J., Hirase H., Dragoi G., Buzsáki G. (2003). Organization of cell assemblies in the hippocampus. *Nature*.

[48] Wang J., Ikonen S., Gurevicius K., van Groen T., Tanila H. (2002). Alteration of cortical EEG in mice carrying mutated human APP transgene. *Brain Research*.

[49] Jyoti A., Plano A., Riedel G., Platt B. (2010). EEG, activity, and sleep architecture in a transgenic A*β*PP swe/PSEN1A246E Alzheimer's disease mouse. *Journal of Alzheimer's Disease*.

[50] Scott L., Feng J., Kiss T. (2012). Age-dependent disruption in hippocampal theta oscillation in amyloid-*β* overproducing transgenic mice. *Neurobiology of Aging*.

[51] Balleza-Tapia H., Huanosta-Gutiérrez A., Márquez-Ramos A., Arias N., Peña F. (2010). Amyloid *β* oligomers decrease hippocampal spontaneous network activity in an age-dependent manner. *Current Alzheimer Research*.

[52] Cummins T. D. R., Broughton M., Finnigan S. (2008). Theta oscillations are affected by amnestic mild cognitive impairment and cognitive load. *International Journal of Psychophysiology*.

[53] Adaya-Villanueva A., Ordaz B., Balleza-Tapia H., Márquez-Ramos A., Peña-Ortega F. (2010). Beta-like hippocampal network activity is differentially affected by amyloid beta peptides. *Peptides*.

[54] Shin J. (2010). Theta rhythm heterogeneity in humans. *Clinical Neurophysiology*.

[55] Shin J., Kim D., Bianchi R., Wong R. K. S., Shin H. S. (2005). Genetic dissection of theta rhythm heterogeneity in mice. *Proceedings of the National Academy of Sciences of the United States of America*.

[56] Nerelius C., Sandegren A., Sargsyan H. (2009). *α*-helix targeting reduces amyloid-*β* peptide toxicity. *Proceedings of the National Academy of Sciences of the United States of America*.

[57] Peña-Ortega F., Solis-Cisneros A., Ordaz B., Balleza-Tapia H., López-Guerrero J. J. (2012). Amyloid beta 1-42 inhibits entorhinal cortex activity in the beta-gamma range: role of GSK-3. *Current Alzheimer Research*.

[58] Wisor J. P., Edgar D. M., Yesavage J. (2005). Sleep and circadian abnormalities in a transgenic mouse model of Alzheimer's disease: a role for cholinergic transmission. *Neuroscience*.

[59] Akay M., Wang K., Akay Y. M., Dragomir A., Wu J. (2009). Nonlinear dynamical analysis of carbachol induced hippocampal oscillations in mice. *Acta Pharmacologica Sinica*.

[60] Verret L., Mann E. O., Hang G. B. (2012). Inhibitory interneuron deficit links altered network activity and cognitive dysfunction in Alzheimer model. *Cell*.

[61] Hermann D., Both M., Ebert U. (2009). Synaptic transmission is impaired prior to plaque formation in amyloid precursor protein-overexpressing mice without altering behaviorally-correlated sharp wave-ripple complexes. *Neuroscience*.

[62] Cramer P. E., Cirrito J. R., Wesson D. W. (2012). ApoE-directed therapeutics rapidly clear *β*-amyloid and reverse deficits in AD mouse models. *Science*.

[63] Wesson D. W., Borkowski A. H., Landreth G. E., Nixon R. A., Levy E., Wilson D. A. (2011). Sensory network dysfunction, behavioral impairments, and their reversibility in an Alzheimer's *β*-amyloidosis mouse model. *Journal of Neuroscience*.

[64] Colom L. V. (2006). Septal networks: relevance to theta rhythm, epilepsy and Alzheimer's disease. *Journal of Neurochemistry*.

[65] Carrillo-Reid L., Tecuapetla F., Tapia D. (2008). Encoding network states by striatal cell assemblies. *Journal of Neurophysiology*.

[66] Leão R. N., Colom L. V., Borgius L., Kiehn O., Fisahn A. (2012). Medial septal dysfunction by A*β*-induced KCNQ channel-block in glutamatergic neurons. *Neurobiology of Aging*.

[67] Rui Y., Li R., Liu Y. (2006). Acute effect of *β* amyloid on synchronized spontaneous Ca^2+^ oscillations in cultured hippocampal networks. *Cell Biology International*.

[68] Rönicke R., Mikhaylova M., Rönicke S. (2011). Early neuronal dysfunction by amyloid *β* oligomers depends on activation of NR2B-containing NMDA receptors. *Neurobiology of Aging*.

[69] Santos S. F., Pierrot N., Morel N., Gailly P., Sindic C., Octave J. N. (2009). Expression of human amyloid precursor protein in rat cortical neurons inhibits calcium oscillations. *Journal of Neuroscience*.

[70] Fuentealba J., Dibarrart A., Saez-Orellana F. (2012). Synaptic silencing and plasma membrane dyshomeostasis induced by amyloid-*β* peptide are prevented by Aristotelia chilensis enriched extract. *Journal of Alzheimers Disease*.

[71] Görtz P., Opatz J., Siebler M., Funke S. A., Willbold D., Lange-Asschenfeldt C. (2009). Transient reduction of spontaneous neuronal network activity by sublethal amyloid *β* (1-42) peptide concentrations. *Journal of Neural Transmission*.

[72] Palop J. J., Chin J., Roberson E. D. (2007). Aberrant excitatory neuronal activity and compensatory remodeling of inhibitory hippocampal circuits in mouse models of Alzheimer's disease. *Neuron*.

[73] Roberson E. D., Halabisky B., Yoo J. W. (2011). Amyloid-*β*/fyn-induced synaptic, network, and cognitive impairments depend on tau levels in multiple mouse models of alzheimer's disease. *Journal of Neuroscience*.

[74] Ziyatdinova S., Gurevicius K., Kutchiashvili N. (2011). Spontaneous epileptiform discharges in a mouse model of Alzheimer's disease are suppressed by antiepileptic drugs that block sodium channels. *Epilepsy Research*.

[75] Gurevicius K., Lipponen A., Tanila H. Increased cortical and thalamic excitability in freely moving appswe/Ps1de9 mice modeling epileptic activity associated with Alzheimer's disease.

[76] Brown J. T., Richardson J. C., Collingridge G. L., Randall A. D., Davies C. H. (2005). Synaptic transmission and synchronous activity is disrupted in hippocampal slices taken from aged TAS10 mice. *Hippocampus*.

[77] Peña F., Tapia R. (1999). Relationships among seizures, extracellular amino acid changes, and neurodegeneration induced by 4-aminopyridine in rat hippocampus: a microdialysis and electroencephalographic study. *Journal of Neurochemistry*.

[78] Peña F., Alavez-Pérez N. (2006). Epileptiform activity induced by pharmacologic reduction of M-current in the developing hippocampus in vitro. *Epilepsia*.

[79] Peña F., Bargas J., Tapia R. (2002). Paired pulse facilitation is turned into paired pulse depression in hippocampal slices after epilepsy induced by 4-aminopyridine in vivo. *Neuropharmacology*.

[80] Sepúlveda F. J., Opazo C., Aguayo L. G. (2009). Alzheimer *β*-amyloid blocks epileptiform activity in hippocampal neurons. *Molecular and Cellular Neuroscience*.

[81] Sanchez P. E., Zhu L., Verret L. (2012). Levetiracetam suppresses neuronal network dysfunction and reverses synaptic and cognitive deficits in an Alzheimer's disease model. *Proceedings of the National Academy of Sciences of the United States of America*.

[82] Sanchez-Mejia R. O., Newman J. W., Toh S. (2008). Phospholipase A2 reduction ameliorates cognitive deficits in a mouse model of Alzheimer's disease. *Nature Neuroscience*.

[83] Balleza-Tapia H., Peña F. (2009). Pharmacology of the intracellular pathways activated by amyloid beta protein. *Mini-Reviews in Medicinal Chemistry*.

